# Epidural administration of 2% Mepivacaine after spinal anesthesia does not prevent intraoperative nausea and vomiting during cesarean section: A prospective, double-blinded, randomized controlled trial

**DOI:** 10.1097/MD.0000000000029709

**Published:** 2022-06-30

**Authors:** Takayuki Kita, Kenta Furutani, Hiroshi Baba

**Affiliations:** a Department of Anesthesiology, Uonuma Kikan Hospital, Minami-Uonuma, Niigata 949-7302, Japan; b Department of Anesthesiology, Niigata University Medical and Dental Hospital, Niigata 951-8520, Japan

**Keywords:** cesarean section, combined spinal and epidural anesthesia, mepivacaine, nausea, visceral pain, vomiting

## Abstract

Intraoperative nausea and vomiting (IONV) is a common symptom during cesarean section (CS) delivery causing significant discomfort to patients. Combined spinal and epidural anesthesia (CSEA) can provide both intraoperative anesthesia and postoperative analgesia. During CSEA, it is reasonable to administer local anesthetics to the epidural space before patient complaints to compensate for the diminished effect of spinal anesthesia. Therefore, we hypothesized that intraoperative epidural administration of 2% mepivacaine would reduce the incidence of IONV.

**Methods:**

Patients who were scheduled for elective CS were randomly allocated to 2 groups. Patients and all clinical staff except for an attending anesthesiologist were blinded to the allocation. After the epidural catheter was inserted at the T11–12 or T12–L1 interspace, spinal anesthesia was performed at the L2–3 or L3–4 interspace to intrathecally administer 10 mg of 0.5% hyperbaric bupivacaine. Twenty min after spinal anesthesia, either 5 mL of 2% mepivacaine (group M) or saline (group S) was administered through an epidural catheter. Vasopressors were administered prophylactically to keep both the systolic blood pressure ≥ 80 % of the baseline value with the absolute value ≥ 90 mm Hg and the mean blood pressure ≥ 60 mm Hg. The primary endpoint was the incidence of IONV. The secondary endpoints were degree of nausea, the degree and incidence of pain, and Bromage score.

**Results:**

Ninety patients were randomized, and 3 patients were excluded from the final analysis. There was no significant difference in the incidence of IONV between the groups (58% in group M and 61% in group S, respectively, P = .82). In contrast, the incidence and degree of intraoperative pain in group M were significantly lower compared to group S. In addition, the incidence of rescue epidural administration of fentanyl (18% vs 47%) or mepivacaine (2.3% vs 25%) for intraoperative pain was lower in group M compared to group S.

**Conclusions:**

Our results indicate that epidural administration of 2% mepivacaine 20 minutes after spinal anesthesia does not reduce the incidence of IONV in CS under CSEA. However, intraoperative epidural administration of 2% mepivacaine was found to improve intraoperative pain.

## 1. Introduction

Intraoperative nausea and vomiting (IONV) is a common symptom experienced during cesarean section (CS) delivery and has been found to occur in 30% to 70% of patients in a placebo group undergoing elective CS delivery.^[1–6]^ IONV occurs during regional anesthesia, causes considerable discomfort to the patient, and increases the risk of surgical complications induced by sudden movement. Therefore, prevention of IONV leads to improvement of anesthesia quality and patient safety during CS.

IONV involves multiple etiologies including hypotension, uterus exteriorization, visceral stimulation, and uterotonic agents.^[1,2]^ Neuraxial opioids are commonly used worldwide to reduce the incidence of IONV.^[7]^ Particularly, intrathecal fentanyl has been reported to effectively improve the quality of regional anesthesia.^[1,8–13]^ However, opioid use causes several complications including pruritus ^[7–10,12]^, and accidental opioid overdose.^[14–17]^ In addition, intrathecal fentanyl was reported to cause acute opioid tolerance.^[18]^ Although the benefit of neuraxial fentanyl for prevention of IONV far outweigh these risks, on the occasion when these side effects are not desirable, opioid-free anesthetic management should be considered.

In Japan, combined spinal and epidural anesthesia (CSEA) is widely used during CS.^[19]^ CSEA can provide both intraoperative anesthesia, especially in cases of failed spinal anesthesia^[20]^ or prolonged surgery, and postoperative analgesia.^[21]^ Although epidural anesthesia can cause some complications such as motor block of lower extremities, a previous study demonstrated that postoperative motor block could be avoided by inserting the epidural catheter at a lower thoracic level to provide better postoperative analgesia.^[19]^ Therefore, CSEA is expected to be a useful anesthesia technique for elective CS without remarkable serious adverse side effects.^[22]^

The inhibition of visceral pain reduces the need for parenteral opioids, which leads to reduced incidence of IONV.^[7]^ Hence, preventing visceral pain is important in preventing IONV. Theoretically, sufficient sensory blockade using neuraxial anesthesia can inhibit noxious signal conductions including visceral pain. Additionally, the cerebrospinal fluid (CSF) concentration of a local anesthetic after spinal or epidural anesthesia gradually decreases over time ^[23,24]^ which leads to the reduced effect of local anesthetics to block visceral pain and increased occurrence of IONV towards the end of CS.^[25]^ Therefore, it is reasonable to assume that intraoperative epidural administration of local anesthetics can maintain CSF concentration, which subsequently blocks the conduction of motor and sensory pathways.^[26]^

Accordingly, maintaining the CSF concentration of the local anesthetic may decrease the incidence of IONV by compensating for the diminished effect of spinal anesthesia. In support of our theory, a recent meta-analysis suggested that CSEA could decrease the incidence of IONV.^[27]^ To the best of our knowledge, no randomized controlled trials have been performed to demonstrate the relationship between IONV and intraoperative epidural administration of local anesthetics during elective CS under CSEA. In the present study, we hypothesized that intraoperative epidural administration of 2% mepivacaine during CS would compensate for the diminished effect of spinal anesthesia to prevent the incidence of IONV, leading to better anesthesia in parturients who undergo CS.

## 2. Materials and Methods

This prospective, double-blinded, randomized controlled trial was approved by the Ethics Committee of Uonuma Kikan Hospital (approval number: 30-036). The study was registered with the JMACCT-CTR (registration number: JMA-IIA00398, Principal investigator: Takayuki Kita, date of registration November 27, 2018). After verbal and written orientation regarding the aims, methods, and risks of the study, informed consent was obtained from all participants. The trial was conducted at the Uonuma Kikan Hospital (Minami-Uonuma, Niigata, Japan) from January 2019 to December 2020. This study adhered to the consolidated standards of the reporting trial statement guidelines (CONSORT).

### 2.1. Participants

The study enrolled patients scheduled for elective CS under CSEA. The exclusion criteria were as follows: American Society of Anesthesiologists physical status ≥ 3; emergency CS; patients scheduled for single-shot spinal anesthesia or general anesthesia; neuraxial anesthesia contraindications; coagulopathy; anticoagulation and antiplatelet therapy; BMI > 35 kg/m^2^; lower extremity neurological abnormalities; spinal abnormalities.

### 2.2. Randomization

A research assistant used computer-generated block randomization (block size: 10) to allocate 90 eligible, consecutive patients to the Mepivacaine (group M) or Saline group (group S) in a 1:1 ratio. The randomized allocation sequence was concealed in sealed, prenumbered, and nontransparent envelopes prepared by the research assistant. Throughout the course of the study, patients, nurses, and all clinical staff involved in the surgery, except for the attending anesthesiologists, were blinded to the group assignments.

### 2.3. Study protocol

After standard monitors were attached and the baseline blood pressure was measured in supine position, the patients were placed in either the left or right lateral position. The insertion site of the epidural catheter (Perifix^®^ FX Catheter; B-Braun, Tokyo, Japan) was anesthetized with 1% mepivacaine using a 25-gauge needle. The epidural catheter was advanced 3 to 5 cm into the epidural space at the T11–12 or T12–L1 interspace using a paramedian approach and the loss of resistance technique with normal saline. A test dose of 2 mL 1% mepivacaine was administered through the epidural catheter to detect intrathecal misplacement. After the insertion of the epidural catheter, spinal anesthesia was performed using a 25-gauge Quincke needle (Spinocan^®^; B-Braun, Tokyo, Japan) at the L2–3 or L3–4 interspace to intrathecally administer 10 mg (2 mL) of 0.5% hyperbaric bupivacaine. After spinal anesthesia, the patients were returned to the supine position, and loss of cold sensation using an ice pack as used to ensure an adequate level of sensory block (T6 or above). If the sensory block failed to reach the T6 level before surgery at 10 minutes after spinal anesthesia, 1% mepivacaine was administered through the epidural catheter until the sensory block reached the T6 level.

Vasopressors were administered prophylactically to minimize the impact on IONV; ephedrine 4 mg when heart rate < 80 beats/min or phenylephrine 0.05 mg when heart rate ≥ 80 beats/min was administered intravenously to keep both the systolic blood pressure ≥ 80 % of the baseline value with the absolute value ≥ 90 mm Hg and the mean blood pressure ≥ 60 mm Hg. Additionally, a left uterine tilt was performed if indicated. Infusion of 6% hydroxyethyl starch 130/0.4 (Voluven®; Fresenius Kabi Japan, Tokyo, Japan) was started after the patient arrival and it was administered as fast as possible after spinal anesthesia until the blood pressure stabilized. If SpO_2_ decreased below 95%, oxygen supplementation was provided using a facemask.

Twenty minutes after the spinal anesthesia, 5 mL of 2% mepivacaine (Carbocaine®; Aspen, Tokyo, Japan) (group M) or 5 mL of saline (isotonic sodium chloride solution; Fuso, Osaka, Japan) (group S) was administered through the epidural catheter. If IONV occurred, 10 mg of metoclopramide was administered intravenously; an additional 1 mg droperidol was administered intravenously if metoclopramide was insufficient. If the patient complained of abdominal pain, mepivacaine or fentanyl was administered into the epidural catheter according to the anesthesiologist’s decision. After delivery of the placenta, 5 IU of oxytocin mixed with 250 mL of 5% glucose solution was infused intravenously. Depending on the condition of the uterine contractions, intravenous administration of methylergometrine or additional oxytocin was requested by the surgeon. The Apgar score was recorded 1 and 5 minutes after birth. If the participants complained of uncontrollable discomfort that led to difficulties in the continuation of the surgery, the anesthesiologist switched to general anesthesia and excluded the participant from the final analysis. At the end of surgery, a patient-controlled epidural analgesia device (Coopdech Balloonjector®; Daiken Medical, Izumi, Japan; a background infusion of 4 mL/h, with a 3 mL bolus dose and 30-min lockout interval) with 0.25% levobupivacaine was attached to the epidural catheter for postoperative analgesia.

Immediately after the surgery, a nurse who was blinded to the allocation used a questionnaire to evaluate the participants’ IONV and intraoperative pain sensation. The questionnaire used a 0 to 10 numerical rating scale (NRS) to evaluate the degree of intraoperative nausea and pain with 0 defined as no complaints, and 10 as worst experience. Additionally, the attending anesthesiologist evaluated the level of sensory block using an ice pack and motor block of the lower extremities using the Bromage Score (0, full flexion of the hip, knee, and ankle; 1, impaired hip flexion; 2, impaired hip and knee flexion; 3, unable to flex the hip, knee, or ankle).

A 1 day postoperative examination was performed to evaluate the degree of nausea and pain, the frequency of patient-controlled analgesia (PCA), and the incidence of pain killer use. Patient satisfaction scores (0, least satisfied; 10, most satisfied) for anesthesia were recorded.

### 2.4. Endpoints

The primary endpoint was the incidence of IONV. The occurrence of nausea was defined as an NRS score >0.

The secondary endpoints were the degree of nausea, degree and incidence of pain, and Bromage score. Additionally, data regarding the pre- and postoperative sensory blockade levels and postoperative nausea and pain were collected.

### 2.5. Statistical analysis

Statistical analysis was performed using SPSS version 27 for Windows (IBM, Armonk, NY). Our preliminary retrospective analysis of patients managed by spinal anesthesia in our hospital estimated the incidence of IONV at 40%. We assumed that intraoperative use of epidural anesthesia would reduce the incidence of IONV by 70%. As a result, at least 80 patients were required for a power of 0.8, and a type 1 error of 0.05. Considering an expected dropout rate of 10%, 90 patients were enrolled in the study and were divided equally amongst the 2 groups. Fisher exact test was used to compare the primary endpoint, the number of patients who were administered methylergometrine, and IONV incidence between the 2 groups. Regarding secondary endpoints, the incidence of intraoperative pain was compared using the Fisher exact test. The degree of nausea and pain, and patient satisfaction scores were compared using the Mann–Whitney U test. The sensory blockade level and Bromage score were compared using the chi-square test. Parametric data were compared using the Student *t*-test. Statistical significance was set at *P* < .05.

## 3. Results

**Figure 1.**
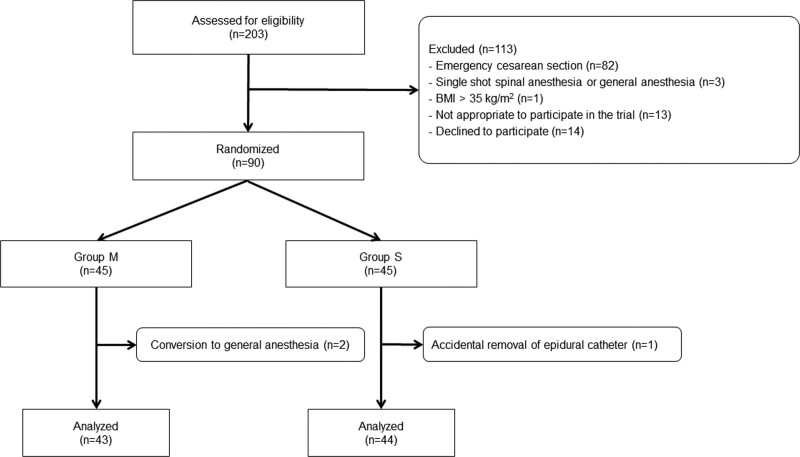
CONSORT flow diagram.

Initially, 203 patients were included in the study; however, 113 patients were excluded. The details of patient selection are shown in Figure 1. A total of 90 patients were included in the randomization; however, 3 were excluded from analysis. Finally, 87 patients were included in the study, 43 in group M, and 44 in group S. The patients’ demographic characteristics and perioperative data are presented in Table 1. No patients received epidural administration of 1% mepivacaine before the surgery due to failed spinal anesthesia. There were no significant differences between groups in total doses of vasopressors and intraoperative infusion volume. Additional methylergometrine was administered for 6 and 2 patients in group M and group S, respectively (*P* = .15).

**Table 1:** Patient characteristics and perioperative data.

	Group M	Group S	*P* value
Age (year)	33 ± 4.6	33 ± 4.5	.81
Height (cm)	157 ± 5.9	158 ± 5.4	.38
Weight (kg)	65 ± 9.9	65 ± 8.8	.89
Gestational age (week)	37 ± 0.9	37 ± 0.8	.55
Operation time (min)	50 ± 16	52 ± 13	.53
Anesthesia time (min)	66 ± 16	69 ± 16	.95
Total ephedrine dose (mg)	10.0 ± 9.3	8.3 ± 7.2	.35
Total phenylephrine dose (mg)	0.24 ± 0.66	0.29 ± 0.58	.70
Total infusion volume (mL)	1058 ± 375	1045 ± 306	.85
Methylergometrine use	6 (13.9)	2 (4.5)	.15
Apgar score at 1 minute	7.8 ± 1.0	7.4 ± 1.4	.13
Apgar score at 5 minutes	8.8 ± 0.5	8.5 ± 0.6	.039

Data are presented as mean ± standard deviation or number (%). P-values are calculated using Student *t*-test or Fisher exact test.

**Figure 2.**
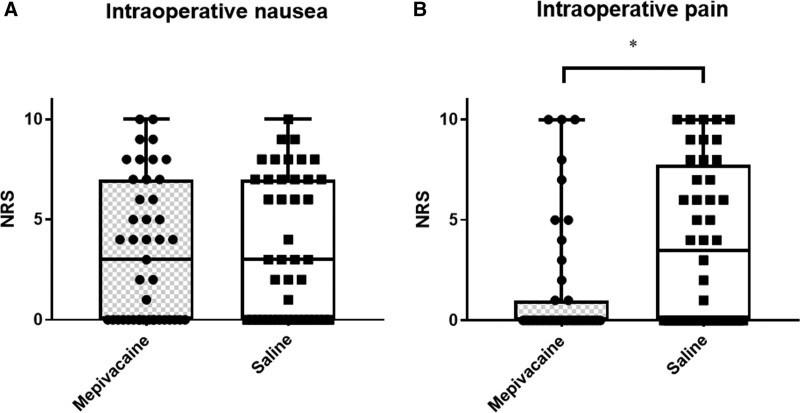
Degree of intraoperative nausea and pain. Box and whisker plots of the NRS of the degree of intraoperative nausea Horizontal lines indicate medians; boxes indicate interquartile ranges; and whiskers indicate ranges. Each dot indicates a value. (A) No significant difference was found regarding the degree of intraoperative nausea between the groups (P=.80, Mann–Whitney U test). (B) The degree of intraoperative pain was significantly lower in group M than in group S (*P = .004, Mann–Whitney U test).

No significant difference was observed in the incidence of IONV (58% in group M and 61% in group S, respectively, *P* = .82, Table 2), degree of nausea (Fig. 2A), and the incidence of rescue antiemetic administration (Table 2) between the 2 groups. Intraoperative vomiting occurred in 1 parturient in each group (Table 2). On the other hand, the incidence and degree of intraoperative abdominal pain were significantly lower in group M compared to group S (Table 2, Fig. 2B). In addition, the incidence of rescue epidural administration of fentanyl (18% vs 47%) or mepivacaine (2.3% vs 25%) for intraoperative pain was lower in group M compared to group S (Table 2). However, no significant difference was observed regarding the Bromage scores and pre- and postoperative sensory blockade levels of the 2 groups (Table 3).

**Table 2:** Intraoperative nausea and pain.

	Group M (n = 43)	Group S (n = 44)	*P*-value
Incidence of intraoperative nausea and pain			
Nausea	58%	61%	.82
Vomiting	2.3%	2.2%	.99
Pain	27%	56%	.009
Incidence of the intervention for intraoperative nausea and pain			
Antiemetic agents	25%	36%	.19
Epidural fentanyl	18%	47%	.006
Epidural mepivacaine	2.3%	25%	.002

*P*-values are calculated using Fisher exact test.

**Table 3:** Data acquired during the cesarean section.

	Group M (n = 43)	Group S (n = 44)	*P*-value
Sensory blockade before surgery			.48
C5	0	1 (2.2)	
T1	4 (9.3)	3 (6.8)	
T2	11 (25)	6 (13)	
T3	6 (13)	8 (18)	
T4	15(34.8)	20(45.4)	
T5	5(11.6)	6(6.81)	
T6	2 (4.6)	0	
Sensory blockade after surgery			.46
T1	4 (9.3)	2 (4.5)	
T2	12 (27.9)	8 (18)	
T3	6 (13.9)	13 (29)	
T4	15 (34.8)	14 (31)	
T5	3 (6.9)	5 (11)	
T6	3 (6.9)	2 (4.5)	
Bromage score after surgery			.16
0	0	0	
1	6 (14)	7 (15)	
2	5 (11)	12 (27)	
3	32 (74)	25 (56)	

Data are presented as number (%). The *P*-values were calculated using the chi-square test.

Postoperative data are shown in Table 4. The incidence and degree of postoperative nausea in group M were significantly higher than those in group S. Additionally, the frequency of PCA usage was significantly higher in group M compared to group S. However, no significant difference was observed regarding the degree of postoperative pain, number of patients who used painkillers postoperatively, and satisfaction score of the patients.

**Table 4:** Postoperative nausea, pain, and patient satisfaction

	Group M (n = 43)	Group S (n = 44)	*P*-value
Nausea	8 (18)	1 (2.2)	.015
Degree of pain (NRS)	3.9 ± 1.9	3.7 ± 2.5	.38
Pain killer use	30 (69)	28 (63)	.65
PCA use (number of times)	12 ± 6.0	9.3 ± 4.9	.011
Patient satisfaction score	7.4 ± 1.9	7.3 ± 2.1	.82

Data are presented as number (%) or mean ± standard deviation.

P-values were calculated using the Mann–Whitney U test or Fisher exact test.

NRS = numerical rating scale, PCA = patient-controlled analgesia.

## 4. Discussion

In the present study, we hypothesized that epidural administration of 2% mepivacaine after spinal anesthesia reduces the incidence of IONV during CS; however, no advantages were found regarding prevention of IONV. In contrast, epidural administration of 2% mepivacaine improved the intraoperative pain score and reduced the dose of epidural rescue fentanyl during CS; this indicates that epidural anesthesia, particularly 5 mL of 2% mepivacaine, cannot reduce the incidence of IONV during CS, even if the epidural anesthesia is effective to improve intraoperative analgesia. Our study failed to demonstrate the superiority of intraoperative administration of mepivacaine through the epidural catheter; however, because our study is a randomized, double-blinded, placebo-controlled trial, the quality of our results should be higher than those of the previous studies. We believe that our results are valuable in that they provide new information regarding whether intraoperative use of epidural anesthesia can prevent the occurrence of IONV during CS.

Although the previous studies^[27–30]^ suggested that CSEA could reduce the incidence of IONV compared with Single-shot spinal anesthesia (SSS), the doses of bupivacaine used for spinal anesthesia were lower in the CSEA group than the SSS group. Another study used the same dose of bupivacaine in both SSS and CSEA group, but only saline was administered through the epidural catheter during CS.^[31]^ In the present study, the same dose of spinal bupivacaine (10 mg) was used in both groups, and the effect of epidural mepivacaine on the occurrence of IONV was compared with the control (saline) group. Therefore, our double-blinded, randomized, placebo-controlled trial provides higher quality evidence than previous studies suggesting that intraoperative epidural administration of mepivacaine was unlikely to reduce the incidence of IONV.

Multiple underlying factors, including anesthetic factors, surgical factors, and uterotonic agents can induce IONV during CS.^[1]^ In the present study, because surgical manipulation and use of the uterotonic agents were common, it is unlikely that these factors affected the results of the present study. Another confounding factor, hypotension induced by blocking sympathetic nerve, is a frequent side effect of neuraxial anesthesia and closely related to the occurrence of IONV. In the present study, hypotension was prophylactically managed by using either ephedrine and/or phenylephrine according to the heart rate to keep the systolic blood pressure ≥ 80 % of the baseline value with the absolute value ≥ 90 mm Hg and the mean blood pressure ≥ 60 mm Hg. As a result, total doses of these vasopressors as well as total volume of infusions were not different between the groups. Therefore, we believe that the incidence of hypotension did not have a significant effect on the results of the present study. In addition, although administration of antiemetic agents such as metoclopramide was known to reduce the incidence of IONV,^[2]^ there were no significant differences in use of antiemetic agents between both groups.

The nonsignificance of our hypothesis may be attributed to the effect of administrating epidural fentanyl as a rescue analgesia. Epidural fentanyl is known to reduce IONV incidence.^[32,33]^ Particularly, intrathecal opioids reduce visceral pain through the inhibition of C-fiber mediated responses.^[34]^ Therefore, neuraxial opioids reduce visceral pain in a dose-dependent manner.^[6]^ In the present study, epidural fentanyl was used as a rescue analgesic for the rescue treatment of intraoperative pain. Consequently, 18% and 47% of patients in groups M and S received epidural fentanyl, respectively. The increased number of patients who received epidural fentanyl could lead to a decrease in IONV, particularly in group S; this may result in the negligible differences between the 2 groups. Additionally, epidural fentanyl use may have affected the incidence of postoperative nausea, which was lower in the group S compared to group M.

Additionally, the dose of local anesthetics may have affected the results. A previous study stated that regional anesthesia mainly blocks Aδ-fibers and local anesthesia may be insufficient to inhibit C-fibers.^[1]^ However, a recent report has shown that bupivacaine inhibits both Aδ-fiber and C-fiber-evoked responses in a dose-dependent manner,^[35]^ especially at high concentrations; therefore, epidural administration of a sufficient dose of a local anesthetic prevents both visceral and somatosensory pain, which can reduce IONV incidence. In this study, 5 mL of 2% mepivacaine was selected due to its early onset of action and avoidance of high epidural anesthesia; however, the dose used may have been insufficient to prevent IONV. A higher dose (concentration, volume, or potency) of a local anesthetic may be necessary if IONV is to be prevented using only epidural anesthesia; this dose will also easily induce hypotension, one of the risk factors for IONV. Because vasopressors were administered prophylactically according to the protocol in both groups, this indicates that the dose of mepivacaine used in the present study has little influence on hemodynamic parameters compared to a placebo.

In the present study, the incidence of IONV in group S (61%) was consistent with previous studies, from 30% to 70% in placebo groups.^[1–6]^ This indicates that we could evaluate IONV correctly as the presence of the intraoperative feeling of nausea/vomiting during CS. Although we assumed that the use of other criteria to define IONV, such as NRS score of ≥5, might affect the results, no significant difference was found in the degree of intraoperative nausea. This suggests that even if mepivacaine can alleviate the degree of intraoperative pain, neither the degree nor the incidence of IONV might be decreased.

## 5. Clinical implication

In the present study, epidural administration of 2% mepivacaine 20 minutes after spinal anesthesia did not affect the incidence of IONV. Our results indicate that it is reasonable for anesthesiologists to use low-dose neuraxial opioids to prevent IONV,^[13]^ even when an epidural catheter is inserted. Although a higher dose of local anesthetic may be necessary to reduce the incidence of IONV without neuraxial opioids, a high dose-local anesthetic increases the risk for hypotension or postoperative motor weakness of the lower extremities. In contrast, the number of patients who required fentanyl as rescue analgesic was evidently lower in group M. Therefore, epidural administration of mepivacaine can improve the intraoperative pain management while decreasing the risk for opioid induced adverse effects or complications.^[7–10,12,14–18,22,36]^ If CSEA is combined with low-dose spinal anesthesia, intraoperative epidural administration of a local anesthetic is a viable alternative strategy in achieving opioid-free anesthesia.^[37]^

## 6. Limitations

The present study had some limitations. First, all outcomes were evaluated immediately after CS. Although it was ideal to evaluate these outcomes during surgery, insufficient human resources limited us in this regard. Second, since our facility is an educational institution, the average surgery time was longer compared to that of other hospitals. As a result, the effect of 2% mepivacaine may have diminished in the latter half of the surgery. Third, the risk factors for IONV were not considered. It has been reported that motion sickness, premenstrual syndrome before pregnancy, and nausea during the first trimester of pregnancy are risk factors for IONV.^[38]^ Fourth, the attending anesthesiologist was not blinded to the allocation to ensure patient safety as well as limited human resource. This could introduce a major risk of bias and may have affected the timing of the anesthesiologist’s administration of rescue analgesics during surgery, especially in the placebo group. The rescue analgesics, especially epidural fentanyl, might reduce the incidence of IONV in group S. Fifth, IONV without requiring any treatments might be included in our results because we strictly defined the occurrence of IONV as NRS > 0. As far as epidural anesthesia was effective to prevent the occurrence of IONV, it would have reduced both the incidence and degree of IONV, even if we had used whichever criteria to assess the incidence of IONV. However, we could not find any differences in those outcomes between the groups. Therefore, we believe that our results can provide clinical significance regarding the limitations of epidural anesthesia. Finally, because epidural mepivacaine was administered intraoperatively to 8 parturients of group S for a rescue analgesic, this might affect the incidence of IONV in group S. However, even if these parturients were included in group M, we could not find any statistical significance in the incidence of IONV between groups (56% in group M and 64% in group S, respectively, *P* = .50, Fisher exact test). Therefore, our data indicate that it would be difficult to prevent IONV using epidural mepivacaine.

In conclusion, our results indicate that epidural administration of 2% mepivacaine 20 minutes after spinal anesthesia could not reduce the incidence of IONV in CS under CSEA. However, it was suggested that intraoperative epidural administration of 2% mepivacaine could improve intraoperative pain and reduce the rescue dose of fentanyl.

## Acknowledgments

We would like to thank all clinical staff at Uonuma Kikan Hospital who helped conduct the present study. We would also like to thank Editage (www.editage.jp) for English language editing.

## Author contributions

All authors contributed to the conception and design of the study. Takayuki Kita and Kenta Furutani performed material preparation, data collection, and analysis. Project administration was performed by Kenta Furutani, and Hiroshi Baba supervised the project. The first draft of the manuscript was written by Takayuki Kita, and all authors commented on the versions of the manuscript. All authors have read and approved the final version of the manuscript.
